# Habitat Use and the Demographics of Object Manipulation by Wild Chacma Baboons

**DOI:** 10.1002/ajpa.70094

**Published:** 2025-07-19

**Authors:** Rassina Farassi, João d’Oliveira Coelho, Susana Carvalho

**Affiliations:** ^1^ Fundació UdG: Innovació i Formació Universitat de Girona Girona Spain; ^2^ Centre for Functional Ecology Universidade de Coimbra Coimbra Portugal; ^3^ Department of Science Gorongosa National Park Chitengo Sofala Mozambique; ^4^ Interdisciplinary Centre for Archaeology and Evolution of Human Behaviour (ICArEHB) Universidade do Algarve Faro Portugal; ^5^ CIBIO, Centro de Investigação Em Biodiversidade e Recursos Genéticos, Campus de Vairão Universidade do Porto Porto Portugal; ^6^ School of Anthropology & Museum Ethnography University of Oxford Oxford UK

**Keywords:** food processing, object use, *Papio ursinus griseipes*, play, problem‐solving

## Abstract

**Objectives:**

Studying object manipulation may offer insights about the emergence of habitual tool use in the hominin clade. Previous research on object manipulation has focused on habitual tool‐using animals such as apes, capuchins, dolphins, and corvids. Investigating object manipulation in wild baboons, a highly social, ecologically adaptable, and terrestrial primate that is not a habitual tool user, can shed further light on the pressures favoring or inhibiting the use of technology. In this study, we investigate factors that influence object manipulation in the chacma baboons of Gorongosa National Park, across demographic and environmental conditions.

**Materials and Methods:**

We collected data using focal and scan sampling, with the aid of the Animal Observer app, and recorded object use and other behaviors. We followed three focal troops: Chitengo, Montebelo, and Floodplain. A total of 2262 observations were recorded across 88 individuals (787 events involved object use).

**Results:**

Mixed‐effects logistic regressions revealed that habitat, age, and substrate use significantly predicted object use among baboons. Object use was most likely in open forests. Adults are less likely to engage in object manipulation, and this behavior decreases with age, which is in line with previous results reported for bonobos. Interestingly, baboons spend more time manipulating objects arboreally than terrestrially.

**Discussion:**

Our findings contribute to the current discussions about the contexts that promote tool use across the primate order. Further studies expanding on these results and assessing differential availability of resources can provide a more comprehensive understanding of the evolution of tool use.

## Graphical abstract

Object manipulation by chacma baboons ( *Papio ursinus* ) in Gorongosa National Park varies by age, sex, and habitat type, with playful use more common in juveniles and food‐focused use in adults, especially in anthropogenic environments.

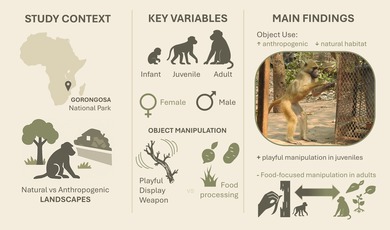

**Summary**

- Object manipulation in chacma baboons influenced by ecological and social factors
- Woodlands favored for object use, while food processing dominates in floodplains.
- Infants and juveniles exhibit higher object manipulation rates than sub‐adults and adults.

## 1 Introduction

Primates have been the focus of many studies in human evolution, particularly our closest relatives, the chimpanzees ( *Pan troglodytes* ) (Carvalho and McGrew 2012), and the more terrestrial, opportunistic, and generalist primates, the baboons (*Papio* sp.) (Carvalho and Beardmore‐Herd 2019; Cowlishaw 2013; Frost et al. 2011). The use of tools and manipulation of objects by non‐human primates has received particular attention, partly due to their close evolutionary relationship with humans (Carvalho and Almeida‐Warren 2019; Haslam et al. 2009; Westergaard 1992), especially in understanding the emergence of technological behaviors in early hominins, who inhabited Africa between 7 and 3 mya (Brunet et al. 2002). However, to date, most of the studies on this topic have focused on great apes given their substantial behavioral and genetic overlap with humans (Haslam et al. 2017; Koops, Furuichi, and Hashimoto 2015; Mazumder and Kaburu 2020; Whiten et al. 1999). While baboons are more distantly related to humans, they exhibit high manipulative ability (Westergaard 1989), large encephalic capacity (Jolly 1970), and spend a considerable amount of time in terrestrial substrates (Frost et al. 2011), offering a broader perspective on the origins of technology in human behavior.

Around the 1960s, non‐human great apes were observed using tools for various purposes, including obtaining food, comfort/hygiene, hunting, and displays of dominance (Goodall 1963; Sanz and Morgan 2013; Whiten et al. 1999). A few years later, “material culture” in chimpanzees and orangutans was announced (Whiten et al. 1999; van Schaik and Pradhan 2003). Another contribution to the study of tool use in non‐human primates was the article by Mercader et al. (2002), where chimpanzees in the Taï Forest in Ivory Coast (West Africa) unintentionally produce flakes during nut cracking, resulting in percussive tool use and organic remains that can be excavated using archaeological methods. It is postulated that repeated use leads to accumulating assemblages or site formations comparable to early hominins (Braun et al. 2025; Mercader et al. 2002). According to Rolian and Carvalho (2017), chimpanzees have the largest known repertoire of tool use and manufacture among primates after humans (McGrew 2017).

Comparative studies of tool use between humans and non‐human primates are shedding light on important questions about the origins of technology. Baboons have been studied since the 1960s as a behavioral model to understand our evolution (Jolly and Whitehead 2000). In terms of taxonomic and ecological classification, the baboon (*Papio* sp.) is a cercopithecoid that diverged from our lineage about 30–32 million years ago, making it a much more distant relative compared to chimpanzees (which diverged ~8 mya) (Kuderna et al. 2023). However, baboons have several characteristics that make them an excellent model species for understanding certain aspects of our evolution. For example, they are the most successful primates in Africa after humans, occupying the greatest quantity and variety of ecological niches (DeVore and Washburn 1963; Jolly and Whitehead 2000). They are successful opportunists (like humans), extreme dietary and habitat generalists, and the most terrestrial of all primates, with a relatively large body size.

Interestingly, baboons are not known to use tools or exhibit behaviors that could be considered cultural. However, very few studies have focused on these aspects and on this genus of primates. A study conducted on chimpanzees ( *Pan troglodytes* ) in Uganda and bonobos ( *Pan paniscus* ) in the DRC (Democratic Republic of Congo) reports that understanding tool use in non‐human primates likely helped identify the conditions that drove the extraordinary expansion of hominine technological advancement. This is because chimpanzees are known for their complex and extensive tool use, while bonobos use very few and none in foraging contexts (Koops, Furuichi, and Hashimoto 2015; Koops, Furuichi, Hashimoto, and Van Schaik 2015).

Wild bearded capuchin monkeys ( *Sapajus libidinosus* ) in Brazil and chimpanzees in West Africa ( *Pan troglodytes verus* ) have been observed deliberately using stones as tools, producing artifacts with striking morphological similarities to early hominin tools. Capuchin monkeys in the Serra da Capivara National Park in Brazil use stone tools for various purposes more extensively than any other known nonhuman primate. They use tools to crush food, dig tubers, crack nuts, process seeds and fruits, and engage in stone percussion (Falótico et al. 2019; Falótico and Ottoni 2013; Musgrave and Sanz 2018). Therefore, certain passive hammering techniques used by capuchins resemble techniques found in the earliest archaeological hominin tool assemblages (Proffitt et al. 2016). Thus, these capuchin data provide new parallel support for understanding stone tool manufacture and the origins of technology in early hominins (Ottoni and Izar 2008).

Another non‐human primate, the Nicobar long‐tailed macaque ( *Macaca fascicularis umbrosus* ), exhibits behaviors associated with object manipulation and tool use in six different behavioral contexts involving various types of objects. Males were found to be engaged in tool use and object manipulation more frequently than females. This study expands existing records of object manipulation and tool‐use behavior in non‐human primates, showing Nicobar monkeys engage in diverse tool‐assisted behaviors and object manipulations across contexts (Mazumder and Kaburu 2020).

Technological behaviors, such as stone tool production, have traditionally been linked to early *Homo*, but recent archaeological findings challenge this view. Evidence from Lomekwi (~3.4 mya, West Turkana, Kenya) and tool use by small‐brained hominins like *Homo floresiensis* suggests that technology predates both the *Homo* genus and significant brain enlargement (Harmand et al. 2015; Carvalho and Beardmore‐Herd 2019; McPherron et al. 2010). Some Australopithecus, including *Australopithecus afarensis*, likely used stone tools as well (Carvalho et al. 2012; Delson et al. 2000). These discoveries imply that tool use may have co‐evolved with other key traits such as bipedalism and access to high‐energy foods like marrow, reshaping our understanding of early technological evolution (Thompson et al. 2019). Ecological opportunity has become a frequently discussed topic among authors investigating wildlife's habitat preferences and mobility patterns (Airola and Barrett 1985; Chapman 1988), particularly among non‐human primates. Therefore, it is believed that these ecological opportunities may influence cultural processes, with resource availability affecting or contributing to habitat selection (Barton et al. 1992; Hamilton III et al. 1978). To determine the environmental factors or conditions involved in wild chimpanzees' foraging tool use (Koops et al. 2013), two ecological hypotheses were tested to explain the prevalence of tool‐use patterns. The opportunity hypothesis posits that encounter rates with resources like nuts, insects, objects, or tools best explain tool‐use patterns. Then, the necessity hypothesis states that tool use responds to the scarcity of preferred foods (Fox et al. 1999, 2004; Koops et al. 2013; Sanz and Morgan 2013). The results from this study support the opportunity hypothesis by revealing that resources, which were rare and peripheral for chimpanzees, played a larger role compared to the necessity to consume them.

Following Hamilton III et al. (1978), we define object manipulation as the purposeful use of any object or substrate, excluding behaviors related to food processing unless they involve the use of a second object (i.e., tool use). This comprehensive definition enables more effective comparisons across troops and populations. According to Hayashi et al. (2006) and Koops, Furuichi, and Hashimoto (2015) object manipulation involves inherent predispositions that initially appear as the ability to interact with a single object using a single action and later develop into the capacity to coordinate multiple actions with multiple objects.

Object manipulation, play, and social learning of tool use in great apes, monkeys, and baboons are often debated, particularly for studying the ontogenetic development of these primates, especially juveniles and infants, based on manipulative behaviors (S. A. S. A. Altmann 1979; Resende & de Resende and Ottoni 2002). These studies generally focus on three recognized types of play: social play involving more than one individual and mainly consisting of chasing or wrestling; locomotor (solitary) play, where an individual jumps and runs alone; and object play, where an individual repetitively manipulates an object (Burghart 1998; Resende & de Resende and Ottoni 2002; Walters 1987).

Object play and manipulation in non‐human primates are widespread, especially among juveniles, and often serve developmental functions related to motor coordination, exploration, and eventual adult foraging skills (Pellis 1991; Hall 1998; Resende & de Resende and Ottoni 2002). Species vary in the frequency and form of object use: for example, chimpanzee juveniles engage more with objects than bonobos (Koops, Furuichi, and Hashimoto 2015), and male vervets and baboons tend to play more socially than females (Mendoza‐Granados and Sommer 1995; Walters 1987). In capuchins, object play begins with simple manipulations and may progress to complex multi‐object behaviors related to extractive foraging (Resende & de Resende and Ottoni 2002). Similarly, infant baboons have been shown to spontaneously use objects to access food in captive settings (Westergaard 1989). These findings suggest that object manipulation may support skill acquisition and cognitive development across species, highlighting the importance of documenting such behaviors and their social context among chacma baboons in Gorongosa.

Thus, considering demographic, ecological, and behavioral contexts, this study aims to investigate the factors influencing object manipulation among chacma baboons ( *Papio ursinus* ) in Gorongosa National Park. The specific objectives are: (1) to examine how age, sex, and habitat type affect the likelihood of general object manipulation; (2) to explore developmental trends in object use across age categories; and (3) to compare food‐processing versus non‐food‐related object use across demographic groups. To guide the analysis, we formulated the following hypotheses: (a) baboons living in human‐modified (anthropogenic) habitats exhibit a higher frequency of object manipulation due to increased availability of both natural and non‐natural materials; (b) object use shifts across development, with younger individuals more frequently engaging in playful and exploratory manipulation; (c) adult males, due to their larger body size and higher energetic demands, are more likely to engage in food‐processing than in non‐food‐related or playful object manipulation.

## 2 Materials and Methods

### 2.1 Study Site and Subjects

**FIGURE 1:**
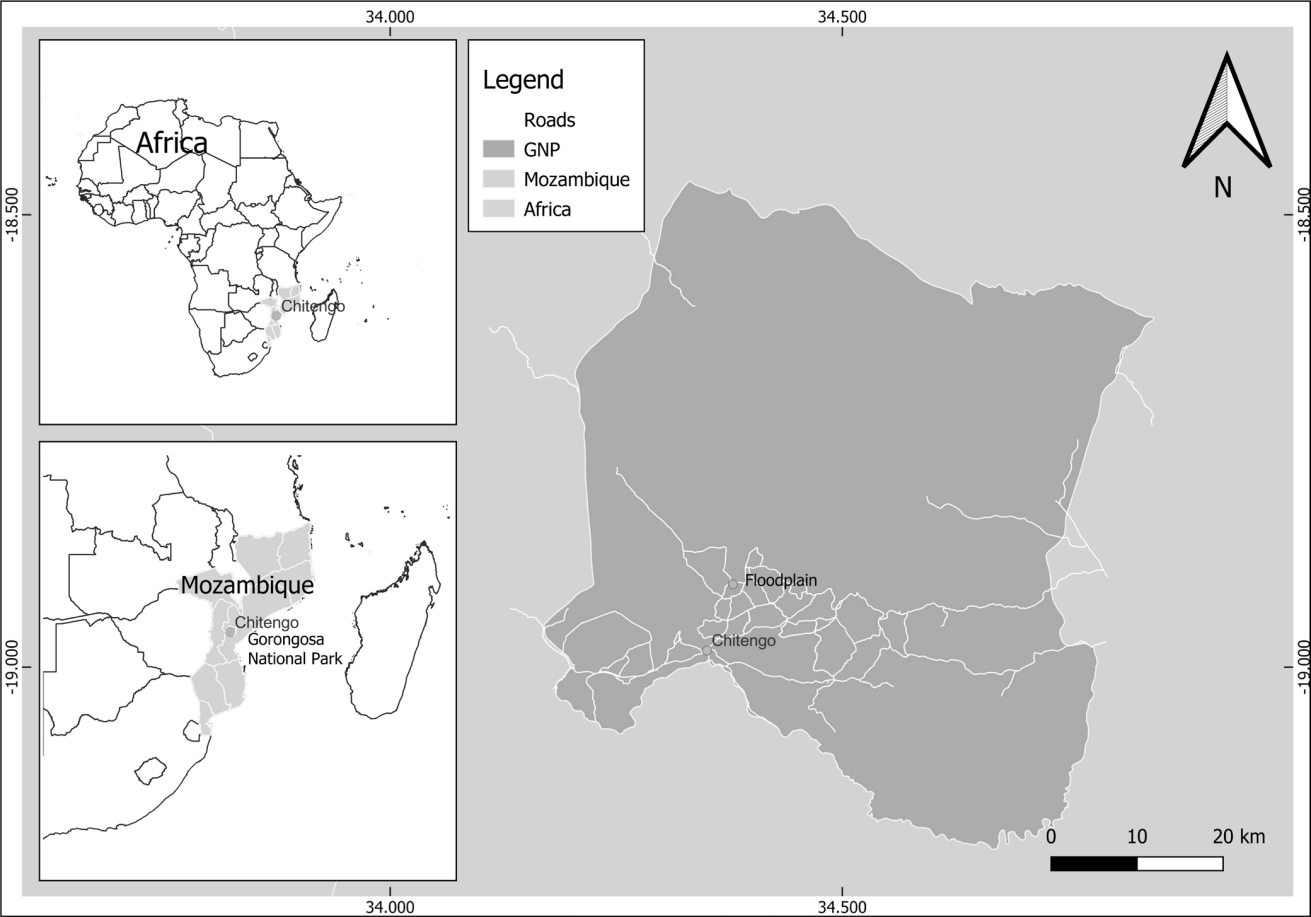
Map showing the location of Gorongosa National Park, Mozambique.

The Gorongosa National Park (18.45° S, 34.45° E) is situated in Mozambique, at the southernmost tip of Africa's Great Rift Valley, one of the continent's most renowned geological features, extending from Ethiopia to the central region of Mozambique in Eastern Africa (Figure 1). The park covers a central area of 4067 km^2^ (inclusive of the Gorongosa mountain range) and extends over 10,000 km^2^, encompassing the buffer zone. Chitengo serves as its primary camp (Hammond et al. 2022).

The seasonal dynamics of the climate strongly influence this region's environmental characteristics and ecological processes. Since savannahs are water‐limited ecosystems, monitoring precipitation trends is crucial for comprehending vegetation changes. According to Herrero et al. (2020), the average annual precipitation in the park from 2000 to 2016 was 1075 mm, with individual annual totals ranging from 665 mm in 2015 to 1415 mm in 2001. The average annual precipitation within the Rift Valley ranges from 700 to 900 mm, with higher precipitation recorded on the valley's flanks. Large areas of the Rift Valley experience seasonal flooding after the peak rainfall in December–February, leading to extensive alluvial plains around the central Urema lake (Stalmans et al. 2019). The annual minimum and maximum temperatures in this region typically range from 15°C to 30°C during the wet and dry seasons, respectively (Herrero et al. 2020).

The park consists of floodplains and *Acacia*‐*Combretum* savannah dominating the Great Rift Valley (Stalmans et al. 2019), while a moist savannah woodland of *Brachystegia* “miombo” occurs at higher elevations to the west (Tinley 1977). Throughout this study, three distinct focal groups were tracked in different habitats: the floodplain troop's home range predominantly encompasses floodplains; however, they return to riverine sleeping sites with denser tree cover (Hammond et al. 2022). Conversely, the Chitengo troop's home range primarily encompasses open savannah woodland. Chitengo, located in the southern part of the park, serves as the primary camp of Gorongosa National Park, where primates have adapted to an anthropogenically altered environment (i.e., with high density of available objects), albeit with mosaic ecological conditions of high vegetation diversity hot spots persisting within an open woodland ecosystem.

Gorongosa baboons were initially documented as gray‐footed chacma baboons ( *Papio ursinus griseipes* ) based on their geographical distribution in Southern Africa (Jolly 2001; Koops et al. 2013; Martinez et al. 2019; Tinley 1977; Zinner et al. 2009). Baboons within the Gorongosa National Park inhabit various groups, with over 200 groups present (Stalmans et al. 2019) within the park and at the Chitengo field base (where two groups have adapted to an anthropogenic environment).

**FIGURE 2:**
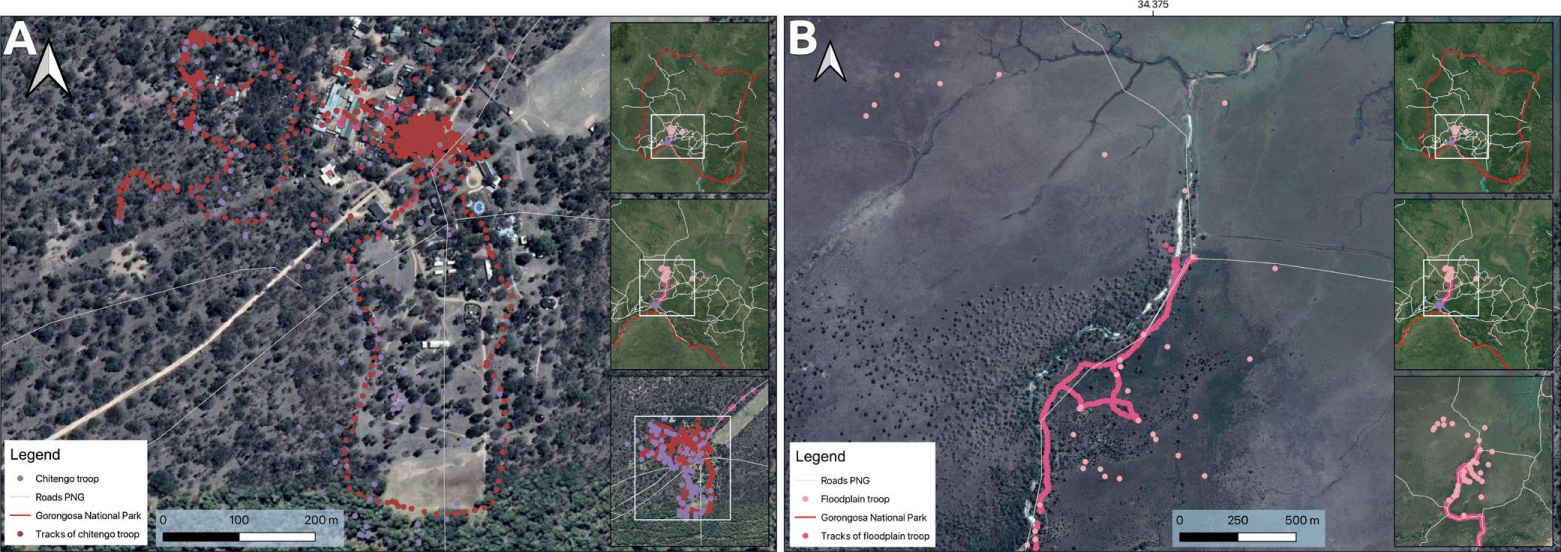
These maps show the home ranges of our study groups. Left shows the Chitengo and Montebelo troops home range in base camp, an area used by humans. The right shows the floodplain troop home range area, where baboons have been studied since 2018 in their natural habitat and without human presence.

The three studied groups (Figure 2) exhibit the following demographic compositions: the Chitengo troop comprises around 45 identified individuals accustomed to human presence, the Montebelo troop includes around 40–50 identified individuals accustomed to human presence, and the Floodplain troop consists of around 40 semi‐habituated identified individuals (see Table 1). We applied the age classification criteria S. A. Altmann (1979) developed to categorize individuals into age groups. We observed 88 focal individuals: 30 adult females, 27 adult males, 4 subadults, 22 juveniles, and 5 infants (see Table 2).

**TABLE 1:** Demographic composition of the identified individuals in the three study troops.

Group ID	Adult males	Adult females	Juvenile males	Juvenile females	Juveniles	Infant females	InfantMales	Total
Chitengo	12	11	0	2	19	0	1	45
Montebelo	12	13	0	1	24	0	0	50
Floodplain	14	15	1	0	8	2	0	40
TOTAL	38	39	1	3	51	2	1	135

**TABLE 2:** Description of the studied individuals from the three baboon troops.

Group ID	Adult	Infant	Juvenile	Subadult	Female	Male	Unknown	Total
Chitengo	23	3	13	2	19	17	5	41
Montebelo	17	1	6	0	11	10	3	24
Floodplain	17	1	3	2	11	7	5	23
TOTAL	57	5	22	4	41	34	13	88

### 2.2 Data Collection

We collected data during two field seasons: from September 13, 2021, to November 25, 2021, and from March 29, 2022, to August 4, 2022, totaling 107 days of fieldwork. During this period, we conducted 214 h of fieldwork, working from Monday to Saturday, from sunrise to sunset, between 07:00–13:00 and 14:00–17:00 daily. There were 197 focal observations, each lasting 10 min, resulting in a total observation time of 1970 min, or approximately 32 h and 50 min, sampled over 57 days. On average, we recorded 3.4 focal observations per day.

We adopted the observation method of scan and focal sampling of individuals (Altmann 1974; Koops, Furuichi, and Hashimoto 2015; Bateson and Martin 2021). Throughout one and a half months, we habituated the three groups again after two years without contact with observers due to the global pandemic. We also tested protocols and collected data using *ad libitum* sampling (Bateson and Martin 2021), accompanied by videos and photos taken with a Nikon P900 camera.

We utilized the “Animal Observer” software program (developed by Damien Caillaud of the Fossey Gorilla Fund International) on an Apple iPad (Tablet 4G) for focal and scan sampling, recording all occurrences of their behavior, taking into account the different categories of behavior, every 10 min throughout the day. However, we only used habitat data to run the models for the scan data. Binoculars were also employed to aid in behavior observation, and trail cameras were used to track the movements of the floodplain group and capture events of “Object Use or Object Manipulation” and “Other Behaviors” for both groups.

**FIGURE 3:**
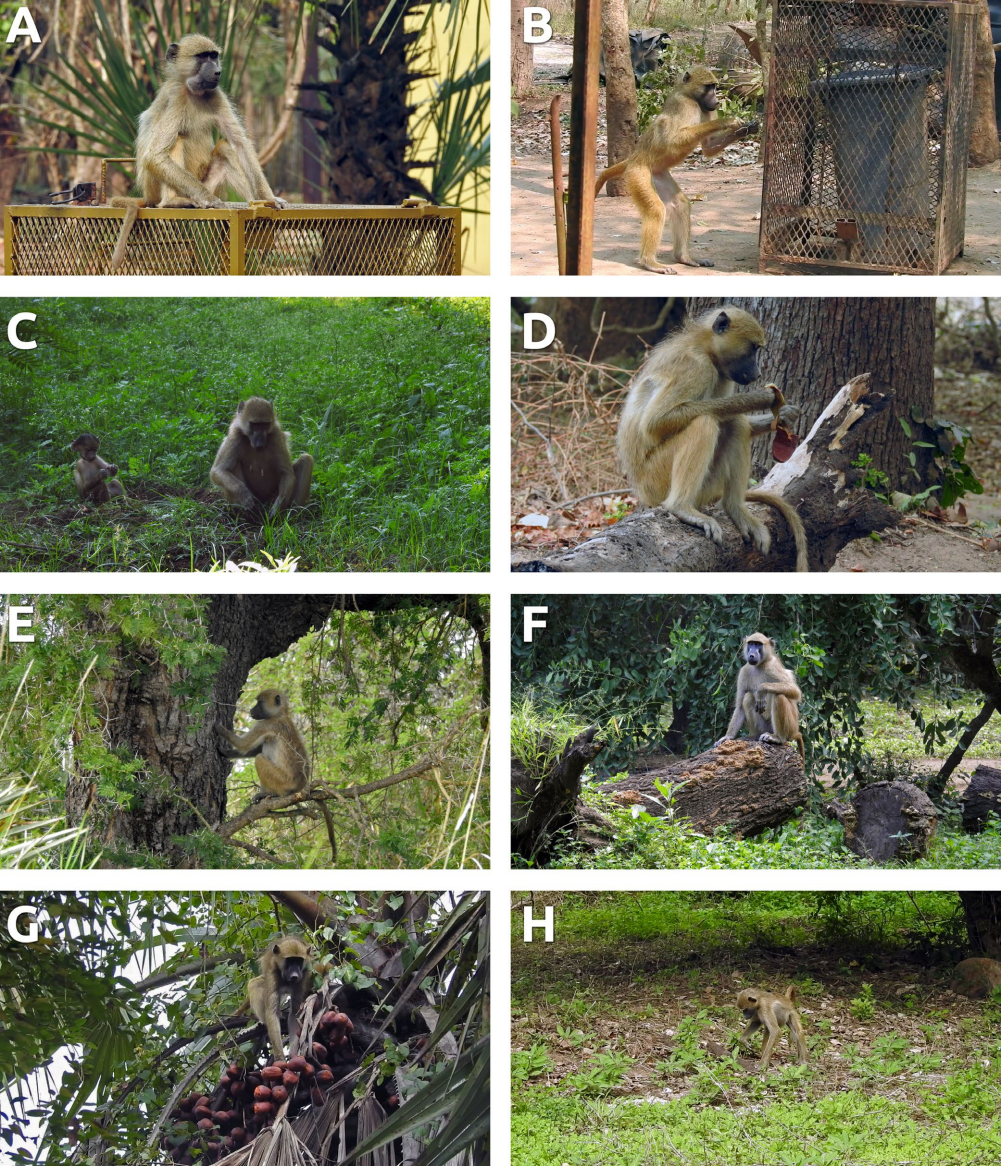
A large male juvenile uses man‐made objects to solve problems/process food, gripping them with both hands (a). An adult male opens and unwinds the wire used to tie the trash bin, point by point, with both hands to achieve a goal/food processing (b). Marge, an adult female with her infant, is digging grass roots/bulbs for their consumption, alternating between both hands (c). A large juvenile uses both hands to process seeds with an apparent goal (d). Another large juvenile uses *Acacia robusta* tree trunks to process food, extracting sap with both hands (e). Chapeu, an adult female, manipulates and uses a tree trunk for display, holding it with her right hand (f). Gabriella, an adult female, manipulates and processes fresh palm fruits with both hands, with an apparent feeding goal (g). A large juvenile manipulates wood and branches in search of dried palm fruit using both hands (h).

We defined an object manipulation event as any instance in which a focal individual actively engaged with a detached or substrate‐anchored object (e.g., sticks, stones, bark, pods, branches, or human debris) using their hands, feet, or mouth, with evident exploratory, extractive, or play‐related actions (e.g., handling, biting, scraping, or striking). This definition excludes routine foraging on food items unless accompanied by non‐feeding manipulations or tool‐assisted feeding (Figure 3). Object manipulation events were scored when the behavior lasted a minimum of 30 s and a maximum of 60 s. This threshold was defined as short events lasting under 30 s were inconsistently visible or ambiguous in function during field conditions. In cases where manipulation clearly continued beyond one minute, the whole bout was recorded as a single discrete event. If the same individual re‐engaged with the same object after placing it down, this was not considered a new event unless a clear temporal break (> 60 s) or a switch in behavioral context was observed (i.e., other type of behavior was recorded in between). Multiple manipulation events could occur during a 10‐min focal follow, and each event was recorded separately.

We recorded both object manipulation and a subset of events we termed object use, defined as instances where the object served an apparent functional role beyond exploratory handling (e.g., play, display, weapon, etc.). Our approach aligns with prior work on object manipulation in nonhuman primates (e.g., Koops, Furuichi, and Hashimoto 2015; Koops, Furuichi, Hashimoto, and Van Schaik 2015), which often use bout‐based scoring of discrete manipulation events per focal period. However, instead of using continuous‐duration measures, our use of discrete events was designed to enhance replicability and reduce observer bias in field conditions with partially obscured views. All focal follows were conducted for 10 min per individual, and each observation was linked to metadata on sex, age class, troop, habitat type, and GPS position (GPSMAP 64st) to mark points and record daily tracks.

Behavioral data were collected using standard continuous focal animal sampling (J. J. Altmann 1974; Bateson and Martin 2021). All observable behaviors of the focal individual were recorded during each 10‐min focal follow, including locomotion, foraging, social interactions, vocalizations, vigilance, object‐related behaviors, and others. Focal individuals were observed at least twice on different days, with sampling evenly distributed across daylight hours to avoid time‐of‐day biases. We recorded object manipulation in relation to seven object types (mollusks, man‐made objects, roots/bulbs, fruits/seeds, leaves, wood, and stones; see Supplementary Tables 1 and 2), and categorized individuals by age, sex, and the availability of manipulable objects in their surroundings.

Similarly, when recording play behaviors or object manipulation, the following parameters were documented: the three groups, Chitengo, Montebelo, and Floodplain; different types of vegetation, namely arboreal (in trees) versus terrestrial (on the ground) and elevated (Supporting Information: Tables 1 and 2); solitary play versus social play; playing with or without objects; vigilance (who is observing whom); manual laterality (which hand is used to manipulate objects); and the function of the manipulated object (whether it is for play/food extraction, weapon/threat, display, scratching the body, etc.) (ethogram in Table 3).

**TABLE 3:** Ethogram utilized during the data collection for the types of object use/manipulation and their definitions observed for each focal individual between September 13, 2021, and November 25, 2021, and from March 29, 2022, to August 4, 2022.

Behavior	Definition
Carrying	Carrying food or infants with hands, feet, or mouth, for example, palm tree nuts
Forage	Foraging without involving object manipulation
Moving	An individual is moving from one place to another, for example, walking or running
Travel	The entire group is moving in another direction, for example, walking or running
Object use	Object use beyond manipulation includes display, weapon, food processing, play, and tool use
Object use: Display	Displaying/shaking branches or twigs with hands directed toward other monkeys, observers, and predators
Object use: Food processing	Problem‐solving through digging for roots and manipulating trash bins to achieve a goal
Object Use: Play	Play involving repetitive movements with an object, without an apparent immediate goal, either alone or with other individuals, for example, jumping on branches
Object use: Weapon	Attacking or aggressing with a falling object or by provoking dust as a threat directed at another individual, observers, and predators, for example, stone
Tool use	Using an object as a tool to achieve a goal, for example, hitting palm fruits with a stone
Play	Playing alone or socially without any object
Rest	Resting without involving any manipulation action
Self‐scratch	It's more than an itch, influenced by an apparent level of stress
Unknown	When we lose sight of the focal individual and cannot observe what they are doing
Vigilant	Patrolling or staying alert if there is any threat or danger, which can be directed at the observer, other monkeys, and predators
Vocalization	Alarm directed at the observer and other monkeys and predators when they feel threatened or in danger
Handedness (Laterality)	Picking up a specific object with the left and right hand or with both hands
Observers	Any individual not playing or manipulating an object observing another individual in activity

Object use events were observed in the following types of vegetation: Floodplain, Sparse woodland (< 1%), Open woodland (1%–10%), Moderate woodland (10%–50%), and Closed woodland (50%–75%) (Supporting Information S1: Tables 1 and 2) (Hammond et al. 2022).

Regarding assessing potential social opportunities for object manipulation, it was postulated to record social interactions of all focal individuals, including the identity of the present social group, to obtain a measure of when they are playing alone or in a group. To assess predispositions for object manipulation, it consisted of recording all actions of manipulation types and the different types of manipulated objects, such as repetitive object play (ethogram in Table 3).

### 2.3 Statistical Analysis I: Object Manipulation Model

To analyze the data, we utilized a mixed‐effects logistic regression model (Ten Have et al. 1998; Vermunt 2005) with the “glmmTMB” package (Brooks et al. 2017) in R v4.0.3 (R Core Team 2023). We collected a dataset comprising a total of 2262 activity observations, of which only 787 events were related to object use. These events were recorded over a total of 214 h (on average: 2 h) and were distributed across three locations: 327 in Chitengo, 150 in Montebelo, and 310 in Floodplain (Figure 2). On average, we recorded 3.456 focal observations per day. For the predispositions of object manipulation and tool use, we initially fitted a model with a binomial distribution using the “logit” link function to model object manipulation rates (as a binomial response). The response variable is thus an object use, with two categories, an object manipulation event (positive class) versus all other activities combined, that is, any other behavior observed, e.g., rest, move, eat, carry, etc. (negative class). The predictor variables of interest included habitat preference (sparse woodland (< 1%), open woodland (1%–10%); moderate woodland (10%–50%); closed woodland (50%–75%); and floodplain), age (infant, juvenile, subadult, and adult), and sex (female, male, and unknown). Additionally, we included the individual ID nested in group ID (Chitengo, Montebelo, and Floodplain) as a random effects variable.

### 2.4 Statistical Analysis II: Object Function Model

In a total of 787 object use events, their function was observed across five categories which consisted of 34 displays, 689 instances of food processing, 53 object play events, and 11 weapon‐related events. We tested whether object manipulation (i.e., display, play, weapon) in a highly terrestrial primate species differed from food processing in terms of ecological and demographic factors. To do this, we constructed a second MELR using a logit distribution to model object function. Given that the majority of object use (function) was described as “food processing,” we grouped the other categories (display, play, weapon) with fewer observations into a binary response variable, classifying them as “object manipulation”. Therefore “object manipulation” (negative class) and “food processing” (positive class) were the only categories of the response variable. The predictor variables included habitat, age, sex, substrate use (ground, raised, and tree), and hand preference (left, right, and both). Additionally, we included the group ID as random effects.

### 2.5 Ethical Note

The research was approved by the Universitat de Girona, Fundació UdG: Innovació i Formació (Reference No: 2020070/002), and the license was granted and authorized by the Ministry of Land, Environment, Rural Development, Administration of Conservation Areas, and the Gorongosa Restoration Project in Mozambique (credential number PNG/DSCi/C210/2021). The research adhered to all protocols established by the Government of Mozambique and the Department of Scientific Services of the Gorongosa National Park. All data collection was purely observational within the three groups in their respective wild habitats, and the researchers/observers never had any physical contact with the monkeys.

## 3 Results

### 3.1 Object Manipulation Model

**FIGURE 4:**
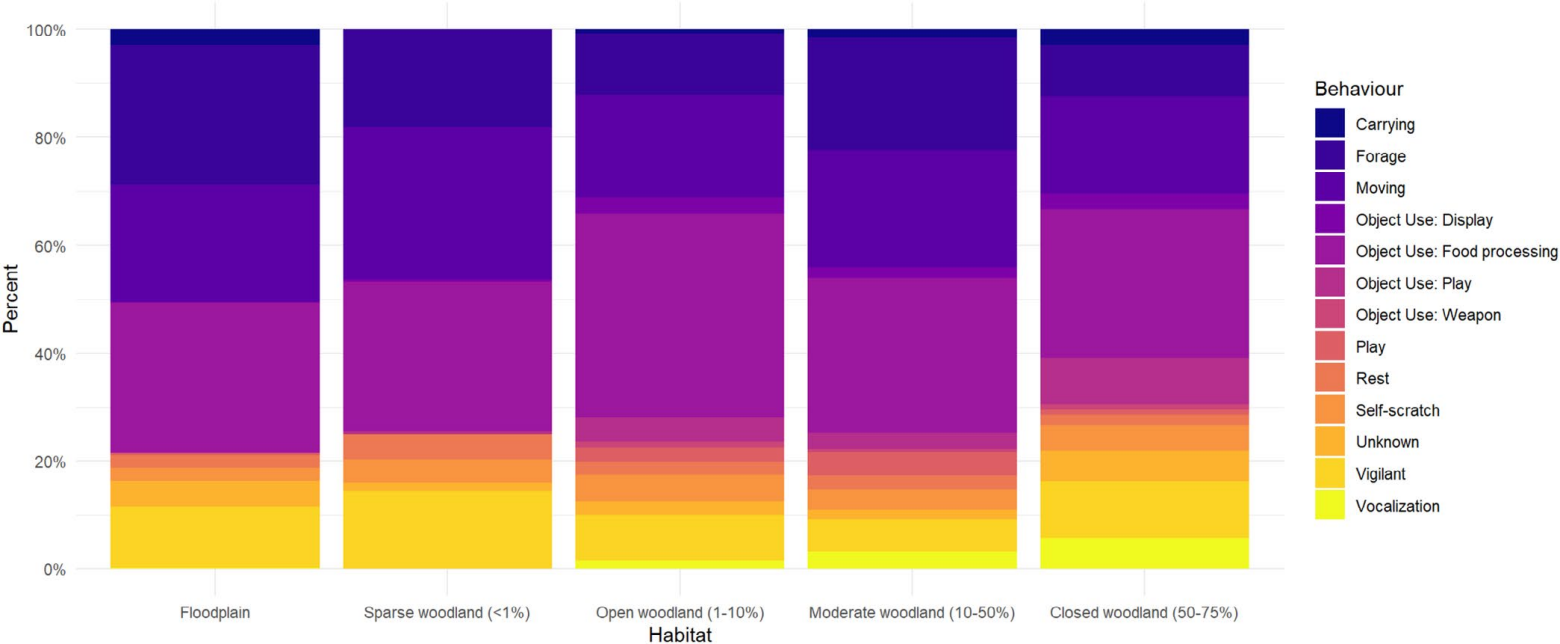
Proportions of observed behavior types in focal individuals for each habitat.

**FIGURE 5:**
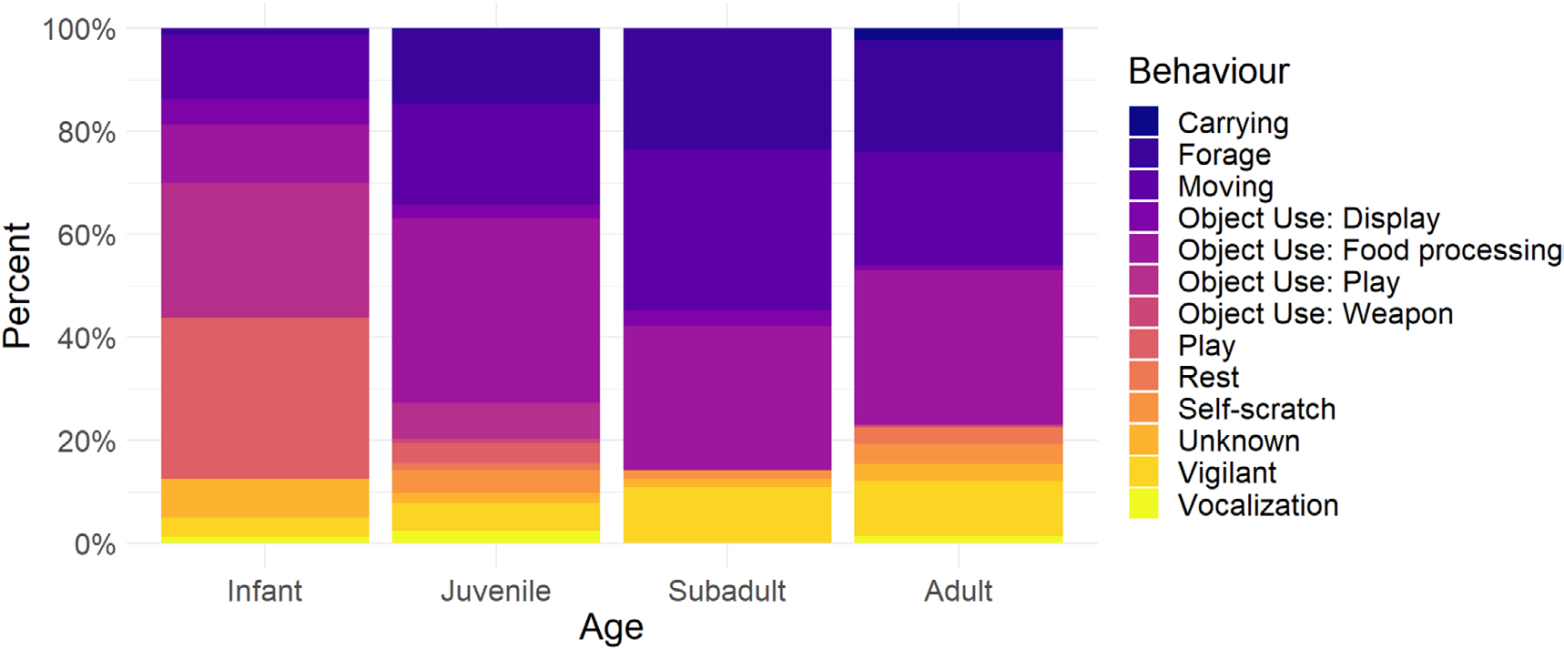
The proportions of behavior/manipulation types for each age class of baboons among the focal individuals (infant, juvenile, subadult, adult).

**FIGURE 6:**
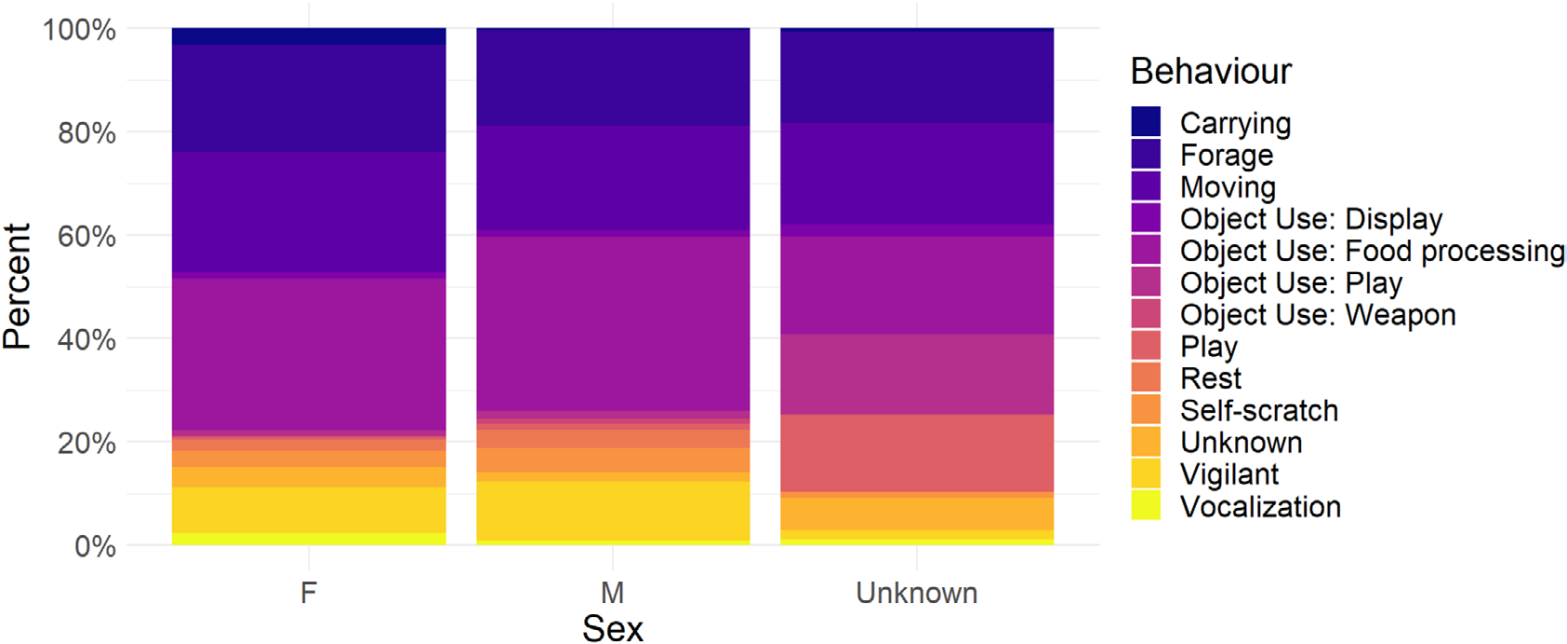
The proportions of behavior/manipulation types for each sex among the focal individuals (sex abbreviated as: F “female” and M “male”).

The MELR model showed that the fixed effects of habitat type, age, and sex significantly predicted the likelihood of object use (Table 4). Habitat type had a significant effect on object use (Figure 4), with individuals in open woodland habitats (1%–10%) being more likely to use objects than those in floodplains (*β* = 0.65, SE = 0.13, z = 5.14, *p* < 0.001). Individuals in closed woodland habitats (50%–75%) also showed a tendency toward increased object use, although the effect was not statistically significant (*β* = 0.41, SE = 0.22, z = 1.84, *p* = 0.065). Age had a negative trend effect on object use (Figure 5), with juveniles being less likely to use objects than infants (*β* = −0.19, not significant) and subadults being even less likely to use objects (*β* = −0.50, not significant); adults were the least likely cohort to engage in object manipulation, and this effect was significant (*β* = −0.67, SE = 0.34, z = −1.97, *p* = 0.048). Sex did not have a significant effect on object use (Figure 6), with males (*β* = 0.02, SE = 0.10, z = 0.20, *p* = 0.85) and unknown (*β* = −0.28, SE = 0.25, z = −1.12, *p* = 0.26) individuals having similar rates of object use to females.

**TABLE 4:** MELR coefficients table of the “Object Manipulation Model”, showing the extrinsic (“habitat”) and intrinsic (“age and sex”) factors influencing object use in chacma baboons.

	Estimate	SE	z value	*p*‐value
(Intercept)	−0.3158980	0.3520495	−0.8973114	0.3695528
Sparse woodland (< 1%)	0.0374919	0.1803871	0.2078416	0.8353526
Open woodland (1%–10%)	0.6502928	0.1265444	5.1388508	**0.0000003**.
Moderate woodland (10%–50%)	0.1762312	0.1224378	1.4393526	0.1500506
Closed woodland (50%–75%)	0.4054718	0.2199866	1.8431664	0.0653047
Juvenile	−0.1938006	0.3234961	−0.5990816	0.5491185
Subadult	−0.5042884	0.4085500	−1.2343369	0.2170774
Adult	−0.6701374	0.3396882	−1.9728013	**0.0485182**
Males	0.0197782	0.1015683	0.1947280	0.8456059
Unknown sex	−0.2790132	0.2500736	−1.1157242	0.2645402

*Note:* Reference categories: Habitat = Floodplain, Age = Infant, Sex = Female. Bold values indicate statistically significant effects (*p* < 0.05).

**FIGURE 7:**
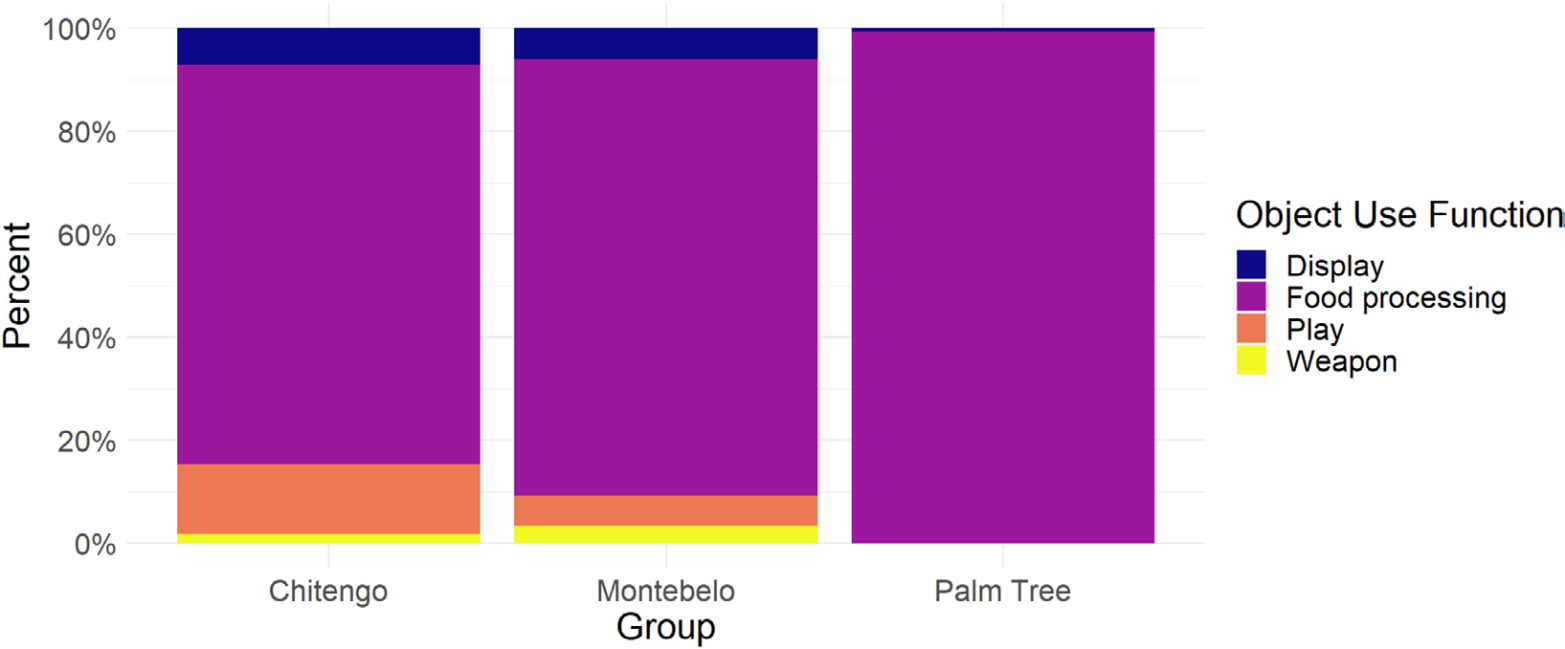
The percentage of object use function types observed for each of the three troops.

### 3.2 Object Function Model

In the second model the dependent variable was a binary variable “Object Function” indicating whether the observed behavior was object manipulation (play, display, and weapons combined; negative class) or food processing (positive class) (Figure 7). The results of the second model are presented in Table 5. Among the predictor variables, Age, Laterality, and Substrate Use had a significant effect on the likelihood of engaging in food processing behavior versus object manipulation behavior. Specifically, adults engaged in significantly less object manipulation and more food processing than infants (*β* = 2.1254, SE = 0.9135, *p* = 0.019). Additionally, when individuals were observed engaging in food processing behavior, they relied more on single‐handed use, particularly with their left hand (*β* = 1.5166, SE = 0.4089, *p* = 0.0002), but they also used their right hand more than both hands combined (*β* = 0.7206, SE = 0.3329, *p* = 0.03). In terms of substrate use, the baboons engaged in significantly more object manipulation when on trees (*β* = −1.7583, SE = 0.4142, *p* < 0.0001) and when raised (*β* = −1.5871, SE = 0.5711, *p* = 0.0055), compared to when on the ground. The effects of the other predictor variables (Habitat and Sex) were not significant in this model. However, the analysis suggests that object use is more frequent in all types of woodland habitats compared to floodplain, while food processing is less frequent in woodland habitats. This is supported by the regression coefficients (*β* ) which range from −0.25 to −2.14, but none were significant. Overall, these results suggest that age and substrate use are important factors influencing the likelihood of engaging in food processing versus other types of object manipulation behavior, and that there may be a hand laterality preference when engaging in food processing behavior.

**TABLE 5:** MELR coefficients table of the “Object Function Model”, comparing the habitats, age, sex, substrate use and laterality variables among focal individuals.

	Estimate	SE	z‐value	*p*‐value
(Intercept)	2.1927918	1.4628234	1.4990133	0.1338702
Sparse woodland (< 1%)	−0.2474461	1.5032331	−0.1646092	0.8692516
Open woodland (1%–10%)	−2.1385693	1.3488516	−1.5854741	0.1128587
Moderate woodland (10%–50%)	−1.7111285	1.3081796	−1.3080226	0.1908656
Closed woodland (50%–75%)	−1.4686820	1.4374074	−1.0217576	0.3068956
Juvenile	1.3536733	0.8274411	1.6359754	0.1018447
Subadult	0.8210687	1.1398719	0.7203166	0.4713301
Adult	2.1253923	0.9135222	2.3265908	**0.0199871**
Males	0.3948042	0.3176932	1.2427215	0.2139705
Unknown sex	−1.0819292	0.7317982	−1.4784528	0.1392866
Raised	−1.5870755	0.5711275	−2.7788461	**0.0054552**
On Tree	−1.7582664	0.4141926	−4.2450458	**0.0000219**
Left hand	1.5166054	0.4088778	3.7091902	**0.0002079**
Right hand	0.7205842	0.3329342	2.1643442	**0.0304379**

*Note:* Reference Categories: Habitat = Floodplain, Age = Infant, Sex = Female, Substrate Use = Ground, Hand Preference = Both. Bold values indicate statistically significant effects (*p* < 0.05).

## 4 Discussion

### 4.1 Object Manipulation Model

Environmental, social contexts, and individual predisposition likely influenced the emergence of technology in hominins (Koops, Furuichi, and Hashimoto 2015). Studying non‐human primate tool use and object manipulation offers a chance to test this hypothesis. Our model explored demographic and environmental factors affecting baboon object manipulation. Results showed habitat type, age, and sex significantly influenced object use. Baboons tended to manipulate objects more in the anthropogenic areas, which in the case of Gorongosa correspond to open forest habitats (1%–10% vegetation cover) of Chitengo, while baboons on the open floodplain were less prone to object manipulation. This is likely because there is a greater density of natural resources and non‐natural objects in the open forest habitats shared by baboons and humans, therefore providing greater opportunities for object manipulation in these areas. Future research should empirically measure object density across different habitats.

Previous studies have shown that ecological opportunity may be an important driver of cultural expression in non‐human primates, particularly regarding the presence, innovation, and perpetuity of tool using behaviors in orangutans, chimpanzees, and capuchins (Carvalho and Almeida‐Warren 2019; Koops et al. 2014). However, cultural differences, like the bonobo‐chimpanzee conundrum, defy pure environmental explanations. Despite both sharing ample ecological opportunities, chimpanzees possess an extensive technological repertoire for extractive foraging, whereas bonobos rarely use tools for such purposes in the wild (Koops, Furuichi, and Hashimoto 2015). In the case of baboons, the present study shows there is a clear link between habitat type and propensity for object manipulation, providing further support to the ecological opportunity model.

Our results also found that age had a significant negative effect on the predisposition for object manipulation. Baboon infants were the most likely to handle objects, with probability rates decreasing through to adulthood. This is consistent with previous findings from bonobos (Koops, Furuichi, and Hashimoto 2015), revealing that adult bonobos were less inclined to manipulate objects when compared to chimpanzees. For both chimpanzees and bonobos, object manipulation rates were higher in contexts of rest and play compared to feeding contexts. In contrast, in our study with baboons, the proportions of object manipulation were greater during feeding and play; we never observed object manipulation during rest. Chimpanzees exhibited higher rates of object manipulation than bonobos, especially in younger individuals of both species, due to limited opportunities for social learning. This pattern was also observed in baboons, which show an ability for object manipulation more similar to chimpanzees than to bonobos. Bonobos also spent more time playing without objects than chimpanzees and baboons. According to Koops, Furuichi, and Hashimoto (2015), these results highlight the crucial role of play in shaping adult behaviors.

Sex differences in object manipulation and tool use have been documented across multiple primate species, often reflecting complex interactions between biological, ecological, and social factors. In macaques and capuchins, for example, males tend to manipulate objects more frequently, potentially due to differences in strength or social opportunities to acquire technical skills (e.g., Mazumder and Kaburu 2020; Falótico and Ottoni 2014). In contrast, female‐biased tool use has been reported in chimpanzees, though patterns vary across populations and behaviors (Lonsdorf 2017; Koops, Furuichi, and Hashimoto 2015; Koops, Furuichi, Hashimoto, and Van Schaik 2015). In this study, biological sex did not have a significant effect on the likelihood of object manipulation in baboons. However, females tended to show a greater propensity for this behavior than males. This tendency echoes previous findings in chimpanzees, where females more frequently and efficiently engage in food‐related tool use, while males are more involved in hunting (Berdugo 2019). Among baboons, males exhibit a stronger inclination toward food processing (*β* = 0.39, *p* = 0.21), consistent with higher activity levels (Owens 1975). These patterns could suggest that object manipulation in non‐adults may serve different developmental functions across sexes.

### 4.2 Object Function Model

The types of objects manipulated by baboons during object use included man‐made objects, roots/bulbs, fruits/seeds, leaves, wood, and stones. None of these object types were used as tools by the three troops during object use. Nevertheless, all these object types have also been observed to be used by chimpanzees, bonobos, orangutans, capuchin monkeys, squirrel monkeys, and long‐tailed macaques (Carvalho et al. 2009; Carvalho and Almeida‐Warren 2019; Koops et al. 2013; Meulman and van Schaik 2013; Sanz and Morgan 2013). This suggests that some of these objects may have been part of the early repertoire of tool use in ancestral humans (Whiten et al. 1999).

The object function model aimed to differentiate food processing from other forms of object use (*sensu* Hamilton III et al. 1978) like display, play, and weapon use. Our results found that the explanatory variables for age, laterality, and substrate use had a significant effect on the probability of an object being used for food processing. Notably, adults were more inclined toward food processing behaviors compared to infants, suggesting a refinement of object manipulation repertoires with age, similar to skill acquisition in technological primates (Figure 6).

Our results also connect with older studies carried out by Westergaard (1989), who reported that captive infant baboons exhibited high variation in the spontaneous use of objects to access food. In contrast, more recent studies by Koops, Furuichi, and Hashimoto (2015) focusing on chimpanzees revealed higher rates of object manipulation for playing than for accessing food resources. Interestingly, infant bonobos present lower rates of object manipulation than infant chimpanzees, albeit they spend more time in the presence of their mothers (Koops, Furuichi, and Hashimoto 2015). In the case of our study, and in line with previous studies, the infant baboons seem to be able to develop this skill independently from social learning opportunities offered by the presence of adult individuals/models (Westergaard 1989). Equally, this may suggest that baboons have a predisposition for object manipulation which does not necessarily lead to the use of tools. Koops, Furuichi, and Hashimoto (2015), Koops, Furuichi, Hashimoto, and Van Schaik (2015) revealed that primates who spend more time playing with objects during their infancy as well as their critical learning periods are more likely to develop tool use behaviors.

With regard to food processing behavior, this has also been described in capuchin monkeys in Brazil, where it was observed in food crushing, tuber excavation, and food processing (Barrett et al. 2018; Falótico et al. 2019; Musgrave and Sanz 2018; Proffitt et al. 2016). On the other hand, it has been noted that object play is more frequently observed and increases as mealtime approaches, while social play decreases in terms of tolerance (Pellis 1991; Resende & de Resende and Ottoni 2002). Within the specific context of food processing, our model found that baboons were more likely to use a single hand, with preference tending to the use of the left hand. Previous captive chimpanzee studies mirrored wild counterparts, revealing individual‐level handedness trends, albeit not population‐wide (Mosquera et al. 2007). Llorente et al. (2009) found consistent hand preference across tasks, hinting at bimanual coordination as a precursor to tool use. Baboons, like chimpanzees, exhibit laterality in object manipulation, underscoring the need for further research to explore this convergence. These findings support the idea that individual‐level lateralization may precede population‐level handedness, a potential precursor to habitual tool use.

Diverse object manipulation was observed among study groups, with the Floodplain troop solely engaging in food processing. Logistical constraints restricted data collection to the dry season for the Floodplain troop, contrasting with the wet season data collection for the Chitengo and Montebelo troops, when resources are more abundant. Nevertheless, these findings complement previous research comparing object manipulation in baboon troops from the Okavango Delta and Namib Desert, which suggested that differences in object manipulation repertoires between troops reflected differences in environmental conditions (Hamilton III et al. 1978). However, it is essential to consider that both Chitengo and Montebelo troops inhabited semi‐natural anthropogenic environments, offering extra chances for interaction with non‐natural objects. Further research is necessary to explore inter‐group differences, the impact of human environments, and potential seasonal variations in object manipulation.

Interestingly, food‐related object manipulation was more common on the ground, while other manipulations occurred more in raised and arboreal settings, possibly due to higher predation risk on the ground (Hammond et al. 2022). Baboons prioritize essential survival behaviors like foraging on the ground, while raised areas offer safer exploration opportunities. Overall, our findings highlight the significance of baboon age and substrate preference in food‐related object manipulation, with a left‐hand bias noted. Differences in manipulation types among troops imply that resource availability and exploration opportunities shape baboon manipulation behaviors.

## 5 Conclusion

Our results indicate that object manipulation rates are influenced by ecological differences, social factors, and predispositions when it comes to using objects and habitat preference. The habitat type most frequently associated with object use was the open woodland, representing the humanized zone closest to Chitengo and Montebelo troop areas. However, the baboons also show a tendency to use objects in more closed habitats. Juveniles display a greater predisposition for object use compared to infants, while adults were the least likely cohort to engage in object manipulation, possibly due to increased time spent foraging. Our study suggests that object manipulation behavior in baboons decreases with aging, and this result aligns more with bonobos than chimpanzees. Our results provide additional information on the ecological and evolutionary conditions that shape object manipulation, which may contribute to the emergence of tool use in primate species. This is a topic that deserves further analysis, particularly in terrestrial, highly encephalized, generalist primates like baboons that share similar environments to early *Homo* and other hominins. In the future, further studies expanding on these results and assessing differential resource availability will provide a better understanding of how baboons develop object manipulation and how it relates to the absence of tool use in the *Papio* genus.

## Author Contributions

**Rassina Farassi:** conceptualization (equal), data curation (lead), formal analysis (equal), funding acquisition (equal), investigation (lead), methodology (equal), project administration (equal), visualization (equal), writing – original draft (equal), writing – review and editing (equal). **João d'Oliveira Coelho:** data curation (supporting), formal analysis (lead), investigation (supporting), methodology (lead), project administration (supporting), supervision (equal), validation (equal), visualization (lead), writing – original draft (equal), writing – review and editing (lead). **Susana Carvalho:** conceptualization (equal), data curation (supporting), funding acquisition (lead), investigation (supporting), methodology (supporting), project administration (equal), resources (lead), supervision (lead), validation (lead), writing – original draft (equal), writing – review and editing (equal).

## Conflicts of Interest

The authors declare no conflicts of interest.

## Supporting information


**Data S1.** Supporting Information.

## Data Availability

The data supporting the findings of this study are available from the corresponding author upon reasonable request. While not publicly archived due to ongoing fieldwork and participant confidentiality considerations, we are happy to share relevant portions of the dataset with qualified researchers for purposes that align with the goals of this study.
