# Supplementary material for: Habitat Use and the Demographics of Object Manipulation by Wild Chacma Baboons
Source: Am J Biol Anthropol. 2025 Jul 19;187(3):e70094. doi: 10.1002/ajpa.70094 (PMC12275083; doi:10.1002/ajpa.70094)
Supplement: Supplementary file 1 — Data S1. Supporting Information. [file AJPA-187-e70094-s001.docx]

**Supplementary Information**

**Appendix/Appendices**

**Table 1** Data collection protocol for chacma baboons (*Papio ursinus griseipes*) in Gorongosa National Park, Mozambique, during two periods: 09/13/2021 to 11/25/2021 and 03/29/2022 to 08/04/2022

| **Activity** | **ObjFunction** | **Obj_Type** | **Obj_N** | **Obj_Mob** | **Obj_Lat** | **Observers** | **Substrate Use** | **Vigilant** | **Vig_At** | **Terrain** |
| --- | --- | --- | --- | --- | --- | --- | --- | --- | --- | --- |
| Object Use | Play | Mollusks | 2 or + | Fixed | Right | Yes | Ground | No |  | Floodplain |
|  |  | Man-made |  |  |  |  |  | Yes | Observer | Sparse woodland (<1%) |
|  |  | Roots/bulbs |  |  | Left |  | Tree | Yes | Monkey | Open woodland (1-10%) |
|  |  | Fruits/seeds | 1 | Mobile |  | No |  |  |  |  |
|  |  | Leaves |  |  | Both |  | Raised | Yes | Predator | Moderate woodland (10-50%) |
|  |  | Woods |  |  |  |  |  |  |  |  |
|  |  | Stones |  |  |  |  |  |  | Others | Closed woodland (50-75%) |
|  | Weapon | Mollusks | 2 or + | Fixed | Right | Yes | Ground | No |  | Floodplain |
|  |  | Man-made |  |  |  |  |  | Yes | Observer | Sparse woodland (<1%) |
|  |  | Roots/bulbs |  |  | Left |  | Tree | Yes | Monkey | Open woodland (1-10%) |
|  |  | Fruits/seeds | 1 | Mobile |  | No |  |  |  |  |
|  |  | Leaves |  |  |  |  |  |  |  |  |
|  |  | Woods |  |  | Both |  | Raised | Yes | Predator | Moderate woodland (10-50%) |
|  |  | Stones |  |  |  |  |  |  | Others | Closed woodland (50-75%) |

**Table 2** Data collection protocol for chacma baboons (*Papio ursinus griseipes*) in Gorongosa National Park, Mozambique, during two periods: 09/13/2021 to 11/25/2021 and 03/29/2022 to 08/04/2022 (continuation)

| **Activity** | **ObjFunction** | **Obj_Type** | **Obj_N** | **Obj_Mob** | **Obj_Lat** | **Observers** | **Substrate use** | **Vigilant** | **Vig_At** | **Terrain** |
| --- | --- | --- | --- | --- | --- | --- | --- | --- | --- | --- |
| Object Use | Weapon | Mollusks | 2 or + | Fixed | Right | Yes | Ground | No |  | Floodplain |
|  |  | Man-made |  |  |  |  |  | Yes | Observer | Sparse woodland (<1%) |
|  |  | Roots/bulbs |  |  | Left |  | Tree | Yes | Monkey | Open woodland (1-10%) |
|  |  | Fruits/seeds | 1 | Mobile |  | No |  |  |  |  |
|  |  | Leaves |  |  | Both |  | Raised | Yes | Predator | Moderate woodland (10-50%) |
|  |  | Woods |  |  |  |  |  |  |  |  |
|  |  | Stones |  |  |  |  |  |  | Others | Closed woodland (50-75%) |
|  | Food processing | Mollusks | 2 or + | Fixed | Right | Yes | Ground | No |  | Floodplain |
|  |  | Man-made |  |  |  |  |  | Yes | Observer | Sparse woodland (<1%) |
|  |  | Roots/bulbs |  |  | Left |  | Tree | Yes | Monkey | Open woodland (1-10%) |
|  |  | Fruits/seeds | 1 | Mobile |  | No |  |  |  |  |
|  |  | Leaves |  |  | Both |  | Raised | Yes | Predator | Moderate woodland (10-50%) |
|  |  | Woods |  |  |  |  |  |  |  |  |
|  |  | Stones |  |  |  |  |  |  | Others | Closed woodland (50-75%) |
|  | Tool Use | Mollusks | 2 or + | Fixed | Right | Yes | Ground | No |  | Floodplain |
|  |  | Man-made |  |  |  |  |  | Yes | Observer | Sparse woodland (<1%) |
|  |  | Roots/bulbs |  |  | Left |  | Tree | Yes | Monkey | Open woodland (1-10%) |
|  |  | Fruits/seeds | 1 | Mobile |  | No |  |  |  |  |
|  |  | Leaves |  |  | Both |  | Raised | Yes | Predator | Moderate woodland (10-50%) |
|  |  | Woods |  |  |  |  |  |  |  |  |
|  |  | Stones |  |  |  |  |  |  | Others | Closed woodland (50-75%) |
